# Demography, Spectrum, and Characteristics of Symptoms Associated With Post-operative Relief After Cholecystectomy

**DOI:** 10.7759/cureus.41171

**Published:** 2023-06-30

**Authors:** Suprabha Shankari, Ajeet P Maurya, Swagata Brahmachari, Pradeep Saxena, Maheshkumar B Jagtap, Sourabh Singh

**Affiliations:** 1 General Surgery, All India Institute of Medical Sciences, Bhopal, Bhopal, IND; 2 Surgery, All India Institute of Medical Sciences, Bhopal, Bhopal, IND

**Keywords:** post-cholecystectomy syndrome, symptoms, cholecystectomy, cholelithiasis, gallstone disease

## Abstract

Background

Cholecystectomy is one of the most commonly performed surgical procedures, and it is indicated for symptomatic gallstone disease. Symptoms of gallstone disease vary; many patients complain of the persistence of symptoms post-operatively. Hence, it is imperative to know the characteristics of symptoms that predict post-operative resolution.

Methodology

A prospective cross-sectional study was performed at a tertiary care centre. Patient demography and pre-operative symptoms were noted. Post-operative persistence or relief of symptoms was also documented. The occurrence of any new symptoms was noted. Data were collected at three and six months after surgery.

Results

Pain was the most common (85%) symptom. The mean frequency of pain was 2.45 per year (range 0-10). The mean duration of pain was 39.7 minutes (range 15-90 minutes). The right hypochondrium (39%) and the epigastric region (42%), along with 8% of patients who experienced pain in both places, were the most frequent locations of pain. The radiation of pain to the right-side scapula is present in 48% of patients.

The pain persisted after one-week follow-up in 28 (34%) of patients, 26 (22%) at the end of one month, and 18 (21%) at the end of six months. Dyspepsia was unresolved in 25%, 20%, and 13% of individuals after one week, one month, and six months, respectively. Upper abdominal discomfort was still persistent in 29%, 26%, and 24% of study subjects at the time of follow-up periods, respectively. Similar persistence is found in symptoms of post-prandial fullness and nausea, where unresolved complaints of post-prandial fullness were present in 18%, 13%, and 10% of patients, respectively, and 26%, 14%, and 10% of patients complained of nausea.

Conclusion

The persistence of symptoms such as upper abdominal discomfort, dyspepsia, post-prandial fullness, and nausea is present, which gradually decreases in severity and presentation over the course of time after the surgical procedure. Some symptoms present after surgery, such as flatulence. Such persistent symptoms might lead to a decreased outcome in terms of patient satisfaction. Patients with atypical pain or dyspepsia need to be counselled pre-operatively regarding the poor resolution of such symptoms.

## Introduction

Gallstone disease is a common condition affecting about 10-15% of the population [1]. There has been a remarkable shift in the trend of gallstone disease from middle-aged, fertile, fat females to young asthenic females in their twenties [2,3]. Gallstones are more common in females, and among elderly males, most people with gallstones remain asymptomatic [4]. Management of gallstones involves cholecystectomy in symptomatic patients [5]. Expectant management is the best approach for patients with incidentally detected, asymptomatic gallstones [6].

Symptomatic cholelithiasis is defined as the presence of gallstones with typical biliary colic pain. However, ‘typical biliary colic’ has been poorly characterized. Upper gastrointestinal motility disorders, gastroesophageal reflux disease, peptic ulcers, and irritable bowel syndrome can have symptoms like gallstones. Non-specific symptoms like nausea, post-prandial upper abdominal discomfort, and dyspepsia may persist after cholecystectomy. Pain and dyspepsia are often the presenting symptoms of post-cholecystectomy syndrome [7]. The common causes of persistent symptoms are peptic ulcers and choledocholithiasis. Functional gastrointestinal disorders also fail to resolve after cholecystectomy. The presence of typical biliary symptoms predicts the relief of symptoms, while atypical symptoms often fail to resolve.

Cholecystectomy carries a significant complication rate and may be deemed unnecessary if symptoms are not attributed to cholelithiasis. The current study was designed to find out the demography and factors predicting the relief of symptoms after laparoscopic cholecystectomy to enable us to better counsel the patients pre-operatively.

## Materials and methods

Patients

The study was performed at a tertiary care centre in central India. The study was a prospective cross-sectional survey. The objectives of the study were to characterize the symptoms associated with relief after surgery and to find out the nature and incidence of new symptoms following cholecystectomy. Consecutive patients (>18 years of age) undergoing laparoscopic cholecystectomy for uncomplicated symptomatic gallstones between December 2019 and October 2021 were included in the study. Exclusion criteria were open cholecystectomy, complicated gallstone disease, previous upper abdominal surgery, and cholecystectomy done with other procedures. Patient demography and pre-operative symptoms were noted. Complicated gallstone disease was defined as gallstones present either with recurrent biliary pain or with some complications like acute cholecystitis, acute pancreatitis, cholangitis, or jaundice. Open cholecystectomy was excluded as wound complications may be a confounding factor. The institutional ethical committee approved the study. Symptomatic gallstone disease is defined by the presence of gallstones, upper abdominal pain, and/or atypical upper abdominal symptoms like dyspepsia, nausea, vomiting, flatulence, bloating, and heartburn. The diagnosis was confirmed by ultrasonography.

Procedure

Patients were consulted pre-operatively and explained about the research protocol. Laparoscopic cholecystectomy was performed using the standard four ports. The gallbladder was extracted through the epigastric port. After obtaining informed consent, patients’ demography and pre-operative symptoms were noted. A biliary symptom questionnaire was used. A visual analogue score (VAS) was used for pain scoring. Co-morbidities were also recorded. Post-operative persistence or relief of symptoms was also documented. Occurrences of any new symptoms were noted. Data were collected at one week, one month, and six months after surgery. Data were collected through face-to-face and telephone interviews. The minimum follow-up period was six months. Patients with persistent pain post-operatively were evaluated for other possible diagnoses using upper gastrointestinal endoscopy and magnetic resonance cholangiopancreatography (MRCP). The persistence of symptoms was defined by symptoms like those in the pre-operative period with varied severity.

Statistical analysis

Quantitative data were expressed as median and range, while qualitative variables were expressed as numbers (proportions, %). The Chi-Square test or Fisher's exact test evaluated the differences in qualitative variables between various groups. Changes in symptoms over time were analyzed by the Wilcoxon signed rank test. Data were fed into a Microsoft Excel worksheet (Microsoft® Corp., Redmond, WA) and analyzed using the Statistical Package for Social Science (SPSS version 17, IBM Corp., Armonk, NY) statistical software for Windows. The normalcy of the data was tested using the one-sample Kolmogorov-Smirnov test. Paired data comparisons were performed using the paired T-test (parametric) and the Wilcoxon signed rank test (non-parametric). A p-value of <0.05 was considered significant.

## Results

Demography

A total of 100 patients were included during the study period (December 2019 to October 2021). All patients were diagnosed with gallstone disease based on symptoms and ultrasound findings. All patients underwent laparoscopic cholecystectomy, and none required conversion to open. The post-operative period was uncomplicated, with a post-operative stay of three to five days. There were 62 (62%) females and 38 (38%) males (Table 1). The mean age was 48.9 years (range 20-75). Female patients tend to have the disease at a younger age. Half of the study subjects (50%) were in the age group of 35-54 years. Most of the patients (84%) were within the normal range of body mass index (BMI) for the population. Fourteen percent of the patients were obese. Pain was the most common (82%) symptom at presentation. Other common symptoms were dyspepsia or indigestion (52%), upper abdominal discomfort (52%), post-prandial fullness (28%), nausea (46%), vomiting (35%), and flatulence (39%). A history of fever was present in 20% of patients.

**Table 1:** Patients’ demography and characteristics of symptoms

S. No.	Characteristics	N=100
1	Gender (M/F)	38:62
2	Mean age	48.9 years (range 20–75)
3	Symptoms	
	Pain	82%
	Dyspepsia	52%
	Upper abdominal discomfort	52%
	Post-prandial fullness	28%
	Nausea	46%
	Vomiting	35%
	Flatulence	39%
	Fever	20%
4	Characteristics of pain	
	Number of episodes	Single: 23%
		2-4: 26%
	Frequency	Mean 2.45 (1–10) per year
	Duration	Mean 39.7 (15–90) minutes
	Localization	Localized: 61%
		Diffuse: 39%
	Location	Epigastric region: 42%
		Right hypochondrium: 38%
	Radiation to the right scapula	85% (n=82)
5	USG characteristics	
	Number of gallstones	Single: 31%
		Multiple: 69%
	Gallbladder wall thickness	Normal: 85%
		Thickened: 15%
	Pericholecystic fluid	15%
	Common bile duct diameter	Average 3.9 mm

Characteristics of pain

The pain was present in 82% of the study subjects, usually of mild severity. Twenty-six percent of patients presented after two to four episodes of pain, while 23% of patients presented after the first episode of pain. The pain was localized in 61% of cases and diffuse in 39%. The mean frequency of pain was 2.45 per year (range 0-10). The mean duration of pain was 39.7 minutes (range 15-90 minutes). The most common location of pain was the epigastric region (42%), followed by the right hypochondrium (39%), while 8% of patients had pain at both locations. Radiation of pain to the right-side scapula was reported in 48% of patients. The nature of pain was colicky in 54%, pricking in 23%, and stabbing in 15%. In 56% of cases, the pain was precipitated or aggravated by food. Medications for pain were needed in 85% of patients. Twenty-seven percent of patients required hospitalization for pain. The pain was localized in 61% of patients and diffused over the entire abdomen in 39%. Analgesics were required in 85% of pain episodes.

USG characteristics

Multiple gallstones were present in 69%, and 31% had a single stone. The echogenicity of the liver was diffusely raised in 54% of individuals, mildly enhanced in 18% of subjects, and grossly normal in 25% of the studied USG patients. The gall bladder was over-distended in 17% of cases, while it was normal size in the remaining 83% of cases on USG findings. The gall bladder wall was thickened in 15% of patients, while the rest (85%) had normal wall thickness. Pericholecystic fluid was seen in 15% of cases. The common bile duct (CBD) diameter on USG was found to be within the normal range (diameter is 3.9 mm), and a dilated CBD (of diameter 6.6mm) was found in one patient. Intrahepatic biliary radical (IHBR) was grossly normal for 99% of the study population; it was found to be dilated for 1%, though. Dilated IHBR, however, was not associated with jaundice or CBD.

Post-op symptoms

**Figure 1:**
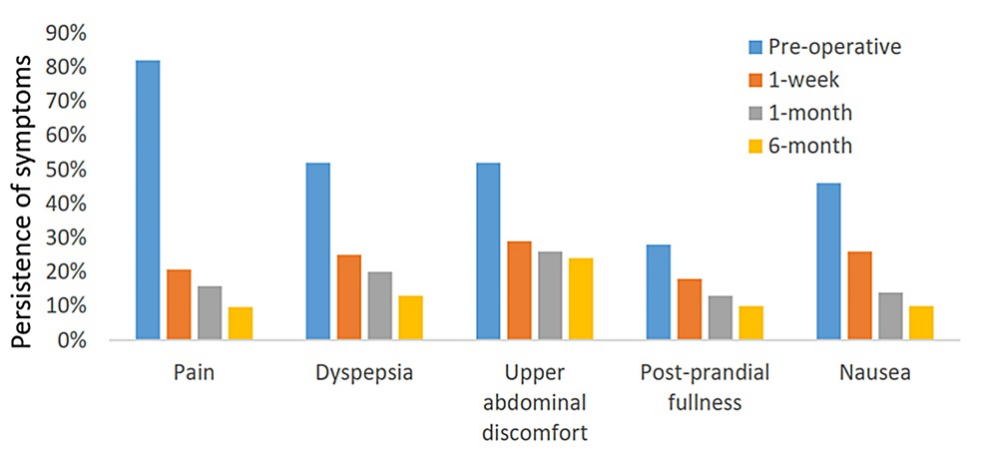
Resolution of symptoms after cholecystectomy

A total of 100 patients took part in a questionnaire regarding demography, symptoms, and follow-up, among whom 82 (82%) patients complained of pre-operative pain. Patients were followed at one week, one month, and six months (Table 2). Persistent pain was reported in 17 (20.7%), 13 (15.8%), and 8 (9.7%) of patients at the end of one week, one month, and six months of follow-up, respectively. Dyspepsia was unresolved in 25%, 20%, and 13% of individuals after one week, one month, and six months, respectively. Upper abdominal discomfort was still persistent in 29%, 26%, and 24% of study subjects at the time of follow-up periods, respectively. Similar persistence is found in symptoms of post-prandial fullness and nausea, where unresolved complaints of post-prandial fullness were present in 18%, 13%, and 10% of patients who complained of nausea, respectively, at the time of follow-up (Figure 1).

**Table 2:** Resolution of symptoms over time

S no.	Symptoms	Pre-operative (n=100)	Post-op follow-up		
			1-week	1-month	6-months
1	Pain	82%	20.7%	15.8%	9.7%
2	Dyspepsia	52%	25%	20%	13%
3	Upper abdominal discomfort	52%	29%	26%	24%
4	Post-prandial fullness	28%	18%	13%	10%
5	Nausea	46%	26%	14%	10%

## Discussion

Gallstone disease affects about 10-15% of the population and is more commonly seen in females. In our study, 62% of patients were female. About 50% of patients were in the 35-55 age group. Although gallstones are more common in obese patients, in our study, 83% of patients had a normal body mass index. A family history of cholelithiasis was noted in 8% of the patients. Pain was the most common symptom. Other common symptoms were dyspepsia or indigestion (52%), upper abdominal discomfort (52%), post-prandial fullness (28%), nausea (46%), and vomiting (35%); 26% of patients presented after two to four episodes of pain. The commonest location of pain was the epigastric area (42%). Radiation of pain to the right scapula was noted in 48% of patients. A similar study found that relief from biliary pain was around 92%, but relief from other symptoms present pre-operatively was low [8]. In our study, at the end of six months, pain persisted in 21% and dyspepsia in 13%.

Post-cholecystectomy syndrome is often caused by choledocholithiasis, gastroesophageal reflux disease, dysfunction of the sphincter of Oddi, remnants of cystic duct stumps, and dropped stones that cause abscesses. Post-cholecystectomy duodenogastric reflux may lead to gastritis [9]. In a prospective study, the following causes of post-cholecystectomy syndrome were reported: no obvious cause (18.4%), Helicobacter pylori infection (15.8%), pancreatitis (15.4%), peptic ulcer disease (15.1%), choledocholithiasis (9.6%), and sphincter of Oddi stenosis (4.4%) [10]. Peptic ulcer disease, gastroesophageal reflux, and hiatal hernias present within three years of cholecystectomy, while choledocholithiasis is present later [11]. Another study reported predictors of persisting symptoms six months after cholecystectomy for patients with different pre-operative symptomatology. At six months, post-operative symptoms were reported by 68.4% of the patients with dyspeptic symptoms, 37.4% of patients with biliary symptoms, and 60.2% of patients with both biliary and dyspeptic symptoms [12]. The presence of functional and anxiety disorders predicts poor relief of symptoms. One study reported a cure for pain in about 90% of patients, and 66% of patients reported complete symptom relief. Symptoms of functional gastrointestinal disorders were present in 88% of patients pre-operatively and were unresolved in 57% postoperatively (P = 0.244) [13]. Flatulent dyspepsia and dull abdominal pain are common presenting symptoms post-operatively. About 50% of patients were not satisfied due to the presence of post-cholecystectomy symptoms [14]. Patients with typical biliary colic have better symptomatic relief, while prolonged pain and flatulence predict poor outcomes [15]. Opioids should be avoided for post-operative pain management as they may prolong the post-operative ileus. In a randomized clinical trial, the combination of intravenous acetaminophen with parecoxib was found to be better than acetaminophen with pethidine or acetaminophen alone [16].

New symptoms like pain, diarrhoea, and flatulence can arise post-operatively. Persistence of pain in up to 33% of the patients and de novo pain in up to 14% were reported in a systematic review. Diarrhoea (85%) and constipation (76%) were the persistent symptoms most often reported, whereas upper abdominal pain and vomiting were the least often reported. Flatulence (62%) was the most often reported new symptom [17]. In our study, the most common persistent symptoms were upper abdominal discomfort (24%), followed by dyspepsia (13%). The other symptoms that persisted after cholecystectomy were post-prandial fullness and nausea. A small sample size and a brief follow-up period were limitations of the current study.

## Conclusions

Pain is reported to be the most common symptom to resolve after cholecystectomy. The severity of persistent symptoms decreases over time. Flatulence is the most often reported new symptom post-operatively. Atypical symptoms should be investigated pre-operatively with further work-up like upper gastrointestinal endoscopy. Such patients should be assured of the benign nature of the problem and treated symptomatically. In the presence of atypical symptoms, pre-operative counselling regarding the possible persistence of symptoms should be done. Patients presenting with atypical symptoms showing stones in imaging studies should also have GERD and IBS ruled out and treated if present to improve the outcome of cholecystectomy when offered.
